# Personalized Computerized Training for Cognitive Dysfunction after COVID-19: A Before-and-After Feasibility Pilot Study

**DOI:** 10.3390/ijerph20043100

**Published:** 2023-02-10

**Authors:** Jon Andoni Duñabeitia, Francisco Mera, Óscar Baro, Tamen Jadad-Garcia, Alejandro R. Jadad

**Affiliations:** 1 AcqVA Aurora Center, Department of Languages and Culture, UiT the Arctic University of Norway, 9019 Tromsø, Norway; 2 Centro de Investigación Nebrija en Cognición (CINC), Facultad de Lenguas y Educación, Universidad Nebrija, 28248 Madrid, Spain; 3 Unidad Long COVID y Síndromes Postvirales, Blue Health Care, 28036 Madrid, Spain; 4 Centro de Salud de Galapagar, 28260 Madrid, Spain; 5 Vivenxia Group, Beverly Hills, CA 90210, USA; 6 Centre for Digital Therapeutics, Toronto, ON M5G 2C4, Canada

**Keywords:** post-acute sequelae of COVID-19, Long COVID, computerized cognitive training, pandemics, digital therapeutics

## Abstract

The current pilot study was set to evaluate the feasibility and potential benefit of a personalized computerized cognitive training (CCT) intervention to improve cognitive function among people living with post-acute sequelae of COVID-19 (PASC). Seventy three adults who self-reported cognitive dysfunction more than 3 months after a diagnosis of COVID-19 took part in an 8-week training study. Participants’ general cognitive function was assessed before they completed as many cognitive daily training sessions as they wished during an 8-week period, using a personalized CCT application at home. At the end of this period, participants repeated the general cognitive function assessment. The differences between the scores at 8 weeks and baseline in five cognitive domains (attention, memory, coordination, perception, reasoning), complemented with analyses of the changes based on the participants’ age, training time, self-reported health level at baseline and time since the initial COVID-19 infection. Participants had significant cognitive dysfunction and self-reported negative health levels at baseline. Most of the participants obtained higher scores after CCT in each of the domains as compared with baseline. The magnitude of this score increase was high across domains. It is concluded that a self-administered CCT based on gamified cognitive tasks could be an effective way to ameliorate cognitive dysfunction in persons with PASC. The ClinicalTrials.gov identifier is NCT05571852.

## 1. Introduction

The coronavirus disease 2019 (COVID-19) pandemic was caused by the pathogen known as Severe Acute Respiratory Syndrome Coronavirus 2 (SARS-CoV-2). Now understood to be a multi-organ illness with a wide range of symptoms, COVID-19 has also resulted in post-acute reports of symptoms and structural organic changes that are extended and chronic, bringing unprecedented levels of additional morbidity. These long-term impacts—known collectively as Long COVID or post-acute sequelae of COVID-19 (PASC) [1]—range from minor complaints to major diseases, reaching even near-fatal situations in some cases, all having a direct impact not only in the physical but also in the psychological health of those affected, and in their levels of productivity [2,3].

PASC comprises a plethora of pulmonary, hematologic, cardiovascular, renal, endocrine, gastrointestinal, dermatologic, neuropsychiatric, and neuropsychological sequelae. Comprehensive large-scale cohort studies have revealed that COVID-19 survivors are at a significantly higher risk of developing neurologic problems than their non-infected peers beyond the first 30 days of infection [4]. One of the most prevalent manifestations of PASC, which affects around one in three individuals, is generalized cognitive dysfunction [2,5,6,7,8]. Although a unified set of diagnostic criteria for PASC is still lacking, there is consensus across major organizations about the inclusion of dysfunctional cognition as a key component [9,10,11]. 

PASC-related cognitive dysfunction (also known as brain fog) is characterized by a range of cognitive indicators, including difficulty with focusing and maintaining attention and memory problems, as well as a general sense of confusion or disorientation [12,13]. The precise mechanisms underlying the relationship between brain fog and Long COVID are not fully understood yet, and further research is needed to clarify this relationship. However, there is little debate on whether brain fog can significantly impact an individual’s quality of life and ability to function, and there is general agreement in that it may be associated with an increased risk of developing more serious cognitive impairments [13].

Despite the knowledge gaps about its root causes, researchers are starting to explore multiple options to curb the consequences of PASC, drawing from studies in populations with similar signs and symptoms emanating from other causes [14]. Along these lines, an option that could be valuable for the management of cognitive dysfunction in people with PASC is personalized computerized cognitive training (CCT), an approach that includes the administration of gamified exercises through digital devices, typically at home. CCTs have been shown to yield significant improvements in dysfunctional cognitive abilities associated with stroke, Parkinson’s disease, age-related cognitive impairment, or multiple sclerosis, among many other conditions, with an intervention duration between 4 and 12 weeks across conditions ([15,16,17,18] for a successful 8-week intervention). Considering this, to the best of our knowledge, we describe what is the first effort to investigate the potential role of CCT to ameliorate cognitive dysfunction in people living with PASC.

Bearing in mind the usefulness of before-and-after feasibility studies with a pre–post design to measure the effectiveness of digital health tools [19], the current study was developed as a first attempt to assess the suitability of a computerized cognitive training by comparing the outcomes before and after the intervention was implemented in a group of individuals with self-reported PASC-related cognitive dysfunction or brain fog. The main objective of this feasibility study was to determine whether the CCT intervention in PASC is practical and feasible to implement in a real-world setting.

## 2. Methods

This was a single-center before-and-after feasibility study set to explore the suitability of a digital health tool based on a CCT for PASC. The details are reported according to the CONSORT extension to pilot and feasibility trials [20]. The study was registered in ClinicalTrials.gov with the identifier NCT05571852. 

### 2.1. Patient and Public Involvement

The design of the current study was led by the CIR Long COVID Research Center, an institution aimed at generating multidisciplinary high-resolution services for people affected by PASC. Once the study finished, the research team organized a meeting with the regional leaders of Long COVID ACTS (Autonomous Communities Together Spain), the main Spanish PASC patient association, in order to share the results in layman’s terms.

### 2.2. Settings, Sampling Frame, and Timelines

The recruitment of the candidate participants was led by the CIR Long COVID Research Center, in collaboration with the Centro de Investigación Nebrija en Cognición (CINC) of Nebrija University (Madrid, Spain). The contact point of Long COVID ACTS disseminated an online message among its affiliates, explaining the study and inviting them to consider enrolling in it. Potential participants sent individual expressions of interest via an online platform specifically designed for this study, which they could access from any place using any device connected to the Internet. Recruitment took place between October 2021 and December 2021, and participants that met the inclusion criteria were accepted on a rolling basis. The first participant completed the study in December 2021 and the last one in February 2022. All phases of the study were conducted online. 

Once eligible participants had been accepted, they completed the cognitive assessments and training in an unsupervised manner. It should be noted, in this regard, that cognitive dysfunction or brain fog is a debilitating manifestation of PASC with different levels of severity that still allow individuals to complete tasks and activities [21].

### 2.3. Selection Criteria and Enrolment

Potentially eligible individuals were included in the study if they: (1) were adults (older than 18 years old), (2) reported having been infected with COVID-19 at least 3 months prior to their expression of interest, and (3) experienced cognitive symptoms associated with PASC (concentration problems or brain fog). A trained neuroscientist from the research team reviewed all self-nominations and identified the candidates who met the selection criteria, excluding those who did not meet them. All participants who met the inclusion criteria signed an informed consent form prior to their enrolment in the study. At that point, their identity was validated by using a procedure based on confirmation emails required to access such a form. Only two exclusion criteria were a priori determined for participants who were accepted into the pilot study: (1) completion of the initial and final cognitive assessment battery, and (2) completion of a minimum of 10 training sessions. No additional constraints were imposed for the sampling, and data from all adult eligible individuals reporting cognitive dysfunction at least 3 months after COVID-19 infection who did not meet the exclusion criteria were analyzed.

### 2.4. Materials and Procedure 

Upon enrolment, included participants completed an online questionnaire with items designed to capture data on their sociodemographic information, their history of infection with COVID-19, and their health status. They were asked to rate their self-perceived estimated percentage of health loss with respect to the time that immediately preceded the COVID-19 infection (options: <25%, 25–50%, 50–75%, >75%), as well as to indicate their self-reported level of health (“In general, would you rate your health as excellent, very good, good, fair or poor?”). Immediately after this, they were asked to complete the Cognitive Assessment Battery (CAB)™ PRO (CogniFit Inc., San Francisco, CA, USA; https://www.cognifit.com/cab). The CAB is a self-administered online general cognitive evaluation psychometric tool [22,23] that takes 30–35 min to complete using either a laptop, desktop, or tablet computer. The CAB includes a series of 17 short tests that evaluate a variety of different cognitive abilities, putting a heavy focus on executive functions. A detailed description of the 17 tests is presented as Supplementary Material. These tests are then used to obtain a gender- and age-adjusted general score, which ranges from 0 to 800 points, as well as five different sub-scores based on the cognitive domains of perception, attention, memory, coordination, and reasoning. The calculation of the cognitive score in each of the five domains is performed by averaging the scores of the individual cognitive skills that constitute them (for reasoning: processing speed, shifting, and planning; for memory: auditory short-term memory, visual short-term memory, short term memory, working memory, visual memory, contextualized memory, naming; for attention: inhibition, focus attention, updating, divided attention; for perception: visual scanning, auditory perception, estimation, recognition, visual perception, spatial perception; and for coordination: response time, eye–hand coordination). The z-score in each of the cognitive domains was obtained for each participant and used as the main outcome measure. These data were obtained using the reference normative dataset of the CAB, which was composed of 1,282,242 unique healthy test-takers (570,980 males and 711,262 females) as of September 2022. 

Once the initial cognitive screening was completed, the digital platform automatically and consecutively assigned participants to the training phase. In this phase, participants were asked to complete short, gamified sessions of around 10 min each, consisting of a variety of tasks specifically designed to tax and train the different cognitive skills. Each training session included two different gamified cognitive tasks selected from a pool of 12 activities. A description of the 12 individual tasks used in the computerized cognitive training is presented as Supplementary Material. Each training program was tailored to the individuals’ specific cognitive strengths and weaknesses detected in the CAB by a patented Individualized Training System™ (ITS) software that automatically chooses the activities and difficulty levels for each person in every session. All individuals were asked to complete a training lasting for 8 weeks in which they could access the training platform as frequently as they desired. The data regarding the adherence of the participants to the training program (as measured by the total number of minutes invested in training sessions) were used as a secondary outcome measure.

After the 8 weeks of training, all participants completed the CAB again and new z-scores in each of the cognitive domains were calculated as compared to the normative sample. 

### 2.5. Data Analysis

The difference in scores between the initial and final assessments was used as the main outcome measure. To this end, the z-transformed scores obtained by each participant in each of the five cognitive domains measured in the CAB before and after the CCT phase (attention, coordination, memory, perception, reasoning) were compared across test moments using both parametric (repeated measures ANOVA) and non-parametric tests (Kruskal–Wallis ANOVA) after exploring whether the data distribution departed from normality with the Shapiro–Wilk test. The ANOVA tests followed a 5 (Domain: attention, coordination, memory, perception, reasoning) by 2 (Test Moment: pre-test, post-test) design. In the presence of a significant interaction, post hoc pairwise comparisons were performed for each level of the Domain factor across the levels of Test Moment. A series of additional ANOVA tests explored the potential mediating role of participants’ age (measured in years; the Age variable) and the time devoted to the training (measured in minutes; the Number of Minutes of Training variable) using them as covariates. Additional analyses explored if their self-reported level of health at baseline (dichotomized into positive health for ‘good’, ‘very good’ or ‘excellent’; or negative for ‘poor’ or ‘fair’; and labeled as the Health at Baseline variable), and the time from COVID-19 infection (dichotomized into less than a year or more than a year from infection; and labeled as the Time from Infection variable) modulated the differences observed between pre- and post-test in each of the five cognitive domains using Fisher’s exact test calculation. Participants’ cognitive assessment scores were also computed in terms of percentiles according to the normative database of the test, and the mean values for each of the cognitive domains were obtained by considering each participant in relation to their corresponding age- and gender-based reference sample. Similarly, the participants’ gender- and age-corrected score in the 0–800 scale generated by the CAB was computed, where scores between 0 and 200 represent a cognitive performance that is well below average, scores between 201 and 400 correspond to a cognitive performance below average, and scores higher than 400 correspond to a cognitive performance above average. The data corresponding to the participants’ sociodemographic profile and their history of COVID-19 infection were analyzed using descriptive statistics. R-based jamovi statistical software was used to run the analysis [24]. In all cases, a *p*-value lower than 0.05 was considered statistically significant. 

## 3. Results

From an initial sample of 262 eligible candidates who volunteered to enter the study, only 103 of them completed the initial and final cognitive assessment (i.e., first exclusion criterion), and 73 of them completed also a minimum of 10 training sessions (i.e., second exclusion criterion). Thus, a total of 73 individuals (mean age = 46.1 years; SD = 7.6; 66 females) were included in the study. While randomized controlled trials typically involve substantially larger sample sizes, the number of participants tested in the current study represents a sample size that provides a sufficient level of confidence for a pilot feasibility study [25]. Two of the participants (2.7%) reported having been infected between 3 and 6 months before their enrolment in the study, 3 individuals (4.1%) between 6 and 9 months before, 10 individuals (13.7%) between 9 and 12 months before, 21 individuals (28.8%) between 12 and 18 months before and 37 individuals (50.7%) more than 18 months before. When asked about how they perceived their current overall health level as compared to the pre-infection stage, 45 individuals (61.6%) reported having lost at least 50% of their health level. Sixty-five participants (89.0%) reported having poor or fair self-rated health at baseline. 

The averaged age- and gender-corrected percentiles associated with the scores obtained in the CAB for the five investigated cognitive domains showed that the test sample was below the median value (50th percentile) across domains at pre-test, pointing to the existence of a generalized cognitive dysfunction (Table 1). Forty-three (58.9%) of 73 participants scored below the 400 cut-off point in attention, 33 (45.2%) in memory, 61 (83.6%) in coordination, 53 (72.6%) in perception, and 50 (68.5%) in reasoning.

The mean number of computerized cognitive training sessions completed across individuals was 51 (SD = 41; median = 44; range: 10–251) and the mean time invested in the intervention was 435 min (SD = 383; median = 358; range: 78–2448). 

There was a consistent increase in the scores obtained in the cognitive assessment after the CCT (i.e., at post-test) as compared with those at baseline, and this increase extended to the five measured cognitive domains (Table 1). Only 25 (34.2%) out of 73 participants scored below the 400 cut-off point in attention, 18 (24.7%) in memory, 40 (54.8%) in coordination, 21 (28.8%) in perception, and 29 (39.7%) in reasoning. The mean percentage of increase in the cognitive score as compared to baseline was a 31% score increase for attention, 37% for memory, 52% for coordination, 42% for perception, and 26% for reasoning. 

When the percentile data calculated after CCT were contrasted with those at baseline, numerical improvements in all the domains were observed (attention: 14 percentile points [95% CI: 10–17]; memory: 18 points [95% CI: 14–21]; coordination: 18 points [95% CI: 14–23]; perception: 17 points [95% CI: 14–21]; reasoning: 11 points [95% CI: 8–15]). Moreover, most of the participants obtained higher absolute scores after CCT in each of the domains, with 59 (81%) achieving improvements in attention, 63 (82%) in memory, 60 (82%) in coordination, 64 (88%) in perception, and 56 (77%) in reasoning scores.

**Figure 1:**
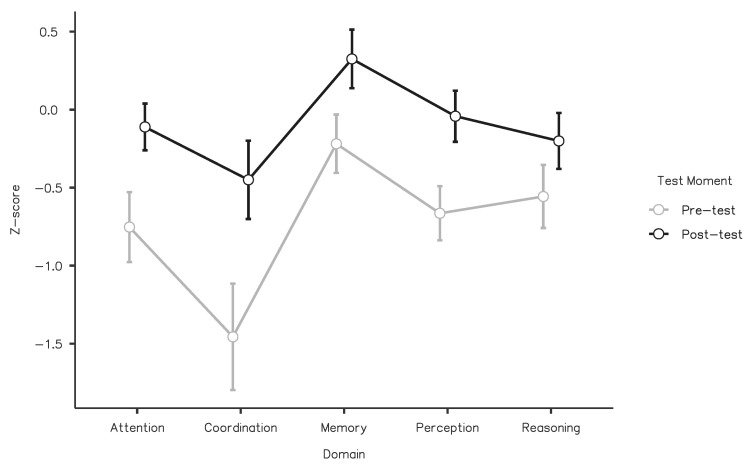
Factorial analysis of z-scores. Error bars correspond to the 95% confidence intervals.

The factorial analysis of the z-scores revealed a significant main effect of Test Moment (F(1,72) = 104.36, *p* < 0.001, η^2^_partial_ = 0.592), suggesting that cognitive performance increased after training (mean difference = 0.63, t(72) = 10.2, Tukey-corrected *p* < 0.001). The interaction between the two factors was significant (F(4,288) = 9.48, *p* <0 0.001, η^2^_partial_ = 0.116), showing that the effect of the training varied as a function of the specific cognitive domain. Post hoc pairwise comparisons showed that scores in all domains improved with training (all ts > 4.25 and Tukey-corrected *p*-values < 0.01), but the mean differences revealed that the gains were not equal across domains (attention = 0.64 [95% CI: 0.47–0.81], coordination = 1.01 [95% CI: 0.75–1.26], memory = 0.54 [95% CI: 0.40–0.69], perception = 0.62 [95% CI: 0.46–0.78], reasoning = 0.36 [95% CI: 0.19–0.52]) (Figure 1).

**Figure 2:**
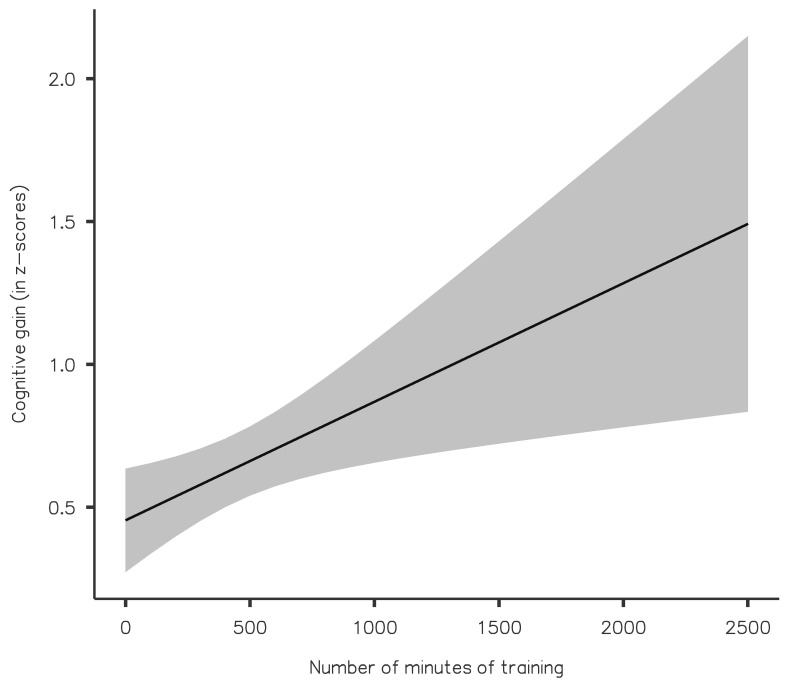
Relationship between minutes of training and cognitive gain (in z-scores). The black line represents the regression line, and the 95% confidence intervals are presented in grey.

The analysis, including the age of the participants and the number of minutes of training invested by each of them as covariates, showed a significant interaction between the Test Moment and the Number of Minutes of Training (F(1,70) = 6.60, *p* = 0.012, η^2^_partial_ = 0.086), indicating that the cognitive improvement increased as a function of the amount of training similarly for all domains (Figure 2). The main effects of Test Moment and Domain, and the interactions between these factors and Age were not statistically significant (all Fs < 1.88 and *p*-values > 0.11). No further analyses were carried out considering the gender factor given that the majority of the sample were female participants, in line with preceding studies and epidemiological data showing a gender bias in Long COVID [26,27].

The examination of the impact of the CCT in each individual cognitive domain as a function of participants’ level of self-reported health at baseline (positive vs. negative) and the time from COVID-19 infection (more vs. less than a year) showed that the existence of cognitive enhancement (presence vs. absence of improvements) did not depend on any of these variables (all *p*-values of the Fisher’s exact tests > 0.21).

Considering that the data were not normally distributed as evidenced by a series of Shapiro–Wilk normality tests (Ws between 0.896 and 0.986, with 8/10 tests being significant at the *p* < 0.05 level), a non-parametric Kruskal–Wallis ANOVA was run to validate the results. All the pre-test vs. post-test comparisons across Domains were significant (attention: χ^2^ = 16.11, *p* < 0.001, ε^2^ = 0.111; coordination: χ^2^ = 16.97, *p* < 0.001, ε^2^ = 0.117; memory: χ^2^ = 16.33, *p* < 0.001, ε^2^ = 0.113; perception: χ^2^ = 27.94, *p* < 0.001, ε^2^ = 0.193; reasoning: χ^2^ = 7.35, *p* < 0.01, ε^2^ = 0.051), endorsing the results of the parametric tests and demonstrating that individuals obtained better cognitive scores after the CCT.

## 4. Discussion

This before-and-after study constitutes the first piece of evidence suggesting that a home-based digital therapeutic program could ameliorate cognitive dysfunction in PASC. Given the potential impact of brain fog on individuals who have had COVID-19 [12], it is important to consider the development of interventions that can help to alleviate these symptoms. The current study suggests that digital therapeutics, which involve the use of software and mobile devices to deliver evidence-based treatments, may offer a promising approach to addressing cognitive dysfunction in the context of Long COVID. The results align with evidence from multiple other conditions involving cognitive dysfunction showing that a CCT yields transfer gains in cognition that can be generalized over time [28]. In addition, this study confirms the findings of previous studies in terms of the severity of the cognitive dysfunction associated with Long COVID, across cognitive domains such as memory, attention, reasoning, or coordination [29,30,31]. The study also provides evidence of long-haulers’ ability to use a digital cognitive evaluation assessment tool to generate a unified general snapshot of their own cognitive status at home.

Still, the findings should be taken with the necessary caution, given the methodological limitations associated with a feasibility before-and-after pilot study, with an unknown common denominator of potentially eligible participants and without a control group. In this sense, some caveats regarding the experimental design need to be highlighted. First, given the nature of this feasibility pilot study, a full neuropsychological or neuropsychiatric assessment of the participants was not carried out. Considering the tight link between problems related to cognitive and mental health and Long COVID [32], future studies should explore the extent to which neurological and psychological effects interact with each other in PASC and describe their contributing role as moderating factors in cognitive interventions. Second, it is worth noting that additional studies with larger samples and the inclusion of control groups are needed to advance our knowledge of the usefulness of a CCT-based intervention for PASC-related cognitive dysfunction. The aim of the current study was to explore if the idea of a CCT in COVID long-haulers was relevant and sustainable, in line with the main objectives of feasibility studies [33]. Considering that the focus was on determining whether the intervention could be implemented and whether it was effective in improving the targeted outcomes, the use of a control group was not deemed necessary. Nonetheless, we acknowledge that randomized controlled trials are required in the near future to compare the outcomes of the intervention group to a group that did not receive the CCT.

Nevertheless, the favorable direction, frequency, and magnitude of the beneficial effects obtained by the participants in this study warrant more rigorous efforts to determine whether a CCT can in fact improve cognitive functions in COVID long-haulers. In particular, such efforts should be conducted under controlled conditions, and include design features to establish the optimal intensity and duration of the intervention, and whether the effects are sustained. To this end, randomized controlled trials allowing for studying generalization and far-transfer effects to other cognitive and psychological domains with both immediate and delayed post-intervention assessments would be useful. 

In conclusion, cognitive dysfunction or brain fog is a common symptom experienced by individuals who have had COVID-19, and it is a generalized manifestation of post-acute sequelae of the disease. The current feasibility study represents the first known step towards developing cost-efficient tele-rehabilitation digital tools to remediate the cognitive dysfunction associated with PASC. Further research is needed to understand the relationship between cognitive dysfunction and Long COVID, and to develop effective interventions for addressing these symptoms. If confirmed, the findings of this study could open the door for non-invasive, non-pharmacological interventions to curb the cognitive dysfunction that is disabling millions of people in the aftermath of the COVID-19 pandemic. 

## Figures and Tables

**Table 1:** Averaged scores and percentiles in each cognitive domain before and after the CCT. Standard deviations are provided in parentheses.

	Pre-Test			Post-Test					
Cognitive Domain	z-Score	Percentile	Score 0–800	z-Score	Percentile	Score 0–800	Participants That Improved	Mean Percentile Increase [95% CI]	Percentage of Improvement
Attention	−0.75 (0.96)	45 (18)	361 (148)	−0.11 (0.64)	59 (18)	472 (141)	59/73	14 [10,11,12,13,14,15,16,17]	31%
Memory	−0.22 (0.80)	48 (21)	384 (170)	0.33 (0.81)	66 (23)	525 (185)	63/73	18 [14,15,16,17,18,19,20,21]	37%
Coordination	−1.46 (1.46)	35 (21)	280 (167)	−0.45 (1.08)	53 (24)	426 (192)	60/73	18 [14,15,16,17,18,19,20,21,22,23]	52%
Perception	−0.66 (0.75)	42 (14)	332 (115)	−0.04 (0.70)	59 (18)	471 (146)	64/73	17 [14,15,16,17,18,19,20,21]	42%
Reasoning	−0.56 (0.87)	44 (19)	348 (149)	−0.20 (0.77)	55 (21)	440 (169)	56/73	11 [8,9,10,11,12,13,14,15]	26%

## Data Availability

All the data obtained in this study are available upon reasonable request to the corresponding author.

## Supplementary Materials

The following supporting information can be downloaded at https://doi.org/10.6084/m9.figshare.21908571 (accessed on 3 December 2022) Description of the 17 tasks comprising the Cognitive Assessment Battery (CAB)™ PRO and of the 12 gamified tasks used in the computerized cognitive training (CCT).
